# Multi-Layered Human Blood Vessels-on-Chip Design Using Double Viscous Finger Patterning

**DOI:** 10.3390/biomedicines10040797

**Published:** 2022-03-29

**Authors:** Elise Delannoy, Géraldine Tellier, Juliette Cholet, Alice M. Leroy, Anthony Treizebré, Fabrice Soncin

**Affiliations:** 1 CNRS/IIS/Centre Oscar Lambret/Lille University SMMiL-E Project, CNRS Délégation Hauts-de-France, 43 Avenue le Corbusier, 59800 Lille, France; elise.delannoy@univ-lille.fr (E.D.); tellier.geraldine@smmil-e.com (G.T.); cholet.juliette@gmail.com (J.C.); alice.leroy3.etu@univ-lille.fr (A.M.L.); 2 Univ. Lille, CNRS, Centrale Lille, Univ. Polytechnique Hauts-de-France, UMR 8520-IEMN-Institut d’Electronique de Microélectronique et de Nanotechnologie, 59000 Lille, France; anthony.treizebre@univ-lille.fr; 3 CNRS, IRL2820, Laboratory for Integrated Micro Mechatronic Systems, Institute of Industrial Science, University of Tokyo, 4-6-1 Komaba, Meguro-ku, Tokyo 153-8505, Japan

**Keywords:** blood vessels, organs-on-chips, inflammation, permeability, BioMEMS, microfluidics

## Abstract

Blood vessel-on-a-chip models aim at reproducing vascular functions. However, very few efficient methods have been designed to address the need for biological replicates in medium- to high-throughput screenings. Here, vessels-on-chip were designed in polydimethylsiloxane-glass chips using the viscous finger patterning technique which was adapted to create channels with various internal diameters inside a collagen solution and to simultaneously seed cells. This method was refined to create blood vessels composed of two concentric, distinct, and closely appositioned layers of human endothelial and perivascular cells arranged around a hollow lumen. These approaches allowed the formation of structurally correct blood vessels-on-chips which were constituted of either only endothelial cells or of both cell types in order to distinguish the vascular barrier reactivity to drugs in the presence or not of perivascular cells. The established vessels showed a tight vascular barrier, as assessed by immunostaining of the adherens junctions, and were reactive to the natural vasopermeant thrombin and to inflammatory cytokines. The presence of perivascular cells markedly increased the tightness of the vascular barrier and lowered its response to thrombin. The design allowed us to simultaneously challenge in real-time several tens of 3D-reconstituted, multicellular blood vessels in a standard multiwell plate format suitable for high-throughput drug screening.

## 1. Introduction

**Figure 1:**
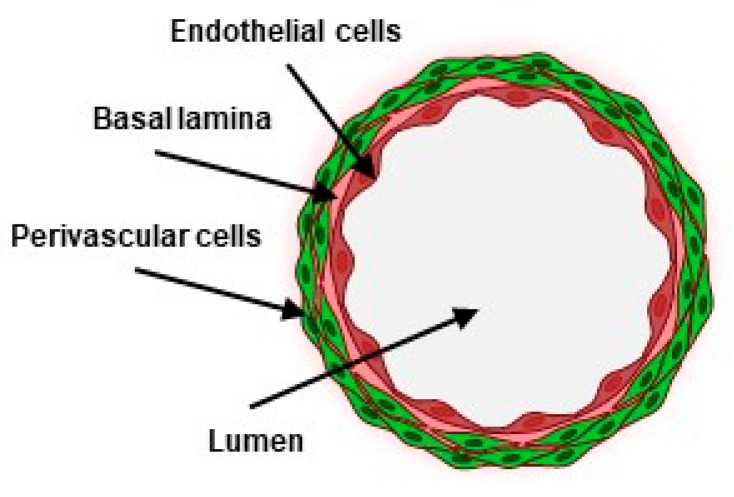
Schematic transversal view of a blood vessel (created with BioRender.com).

Blood vessels form a network of conduits that perfuse blood through most organs in the body. They are structurally composed of concentric layers of vascular cells arranged around a central lumen in which blood circulates. From the inside out, endothelial cells make the inner, most luminal face of blood vessels as a continuous monolayer resting on a thin extracellular matrix. Perivascular cells, such as smooth-muscle cells, are organized as concentric sheets around the endothelium monolayer, from which they are separated by a thin basal lamina (Figure 1). This structural arrangement is essential to the functions of blood vessels, as each cell layer plays specific roles in the vascular barrier. The endothelium is an active and selective barrier to circulating cells and to metabolites. Due to its luminal location, the endothelium also plays essential roles in the regulation of vascular tone, of thrombosis, and of the immune response. Perivascular cells, located at the basal side of the endothelium, provide structural strength, vascular contraction, and make cellular contacts and communications with endothelial cells. Perivascular cells, including pericytes, are essential to the integrity of the endothelium and to the tightness of the vascular barrier [1].

Blood vessels participate in fundamental physiological processes. During inflammation and under the stimulation of pro-inflammatory cytokines, endothelial cells become activated and, consequently, strongly express leukocyte adhesion molecules at their luminal face that favor the rolling, arrest, firm adhesion, and extravasation of immune cells from the circulation toward the tissues [2]. Permeability is another important vascular function which is subsequent to the disruption of endothelial tight and adherens junctions [3]. Vascular permeability is impaired in several pathologies such as inflammation, oedema and ischemic stroke [4,5,6]. In human cancers, weak endothelial cell–cell junctions in the tumor vasculature create gaps and leaky vessels [7] that enable metastatic cancer cells to enter the circulation. Furthermore, the tumor blood vessel endothelium, while being imperfectly tight, actively protects tumor cells from the infiltration of circulating cytotoxic immune cells into the tumor mass through its barrier function [8,9,10,11].

Most current blood vessels-on-chip (VoC) approaches involve soft lithography processes to produce patterns that reproduce the negative imprints of microchannel structures with which polymers such as polydimethylsiloxane (PDMS) are molded [12,13,14]. The microchannels are combined or coated with physiologically relevant hydrogels or matrix molecules, such as collagen or fibronectin, and the surfaces seeded with endothelial cells, in some cases completed with perivascular cells [15]. Few approaches aimed at reconstituting the concentric cell layers of natural blood vessels. In two studies, perivascular cells were randomly seeded into hydrogels surrounding endothelial cell-lined channels [16,17]. In the third one, bone marrow stromal cells were seeded prior to endothelial cells on the same hydrogel surfaces, resulting in composite layers of cells [18]. Although cell contacts and improvement of barrier properties were observed, these models did not reconstitute the typical structural features of natural blood vessels (Figure 1) where the continuous endothelial barrier is surrounded by, but distinct from, perivascular cells. In order to address this structural point-of-view, an alternative approach using removable needles was designed to separately seed endothelial and perivascular cells in tube-like structures [19]. However, this process seemed somewhat cumbersome when aiming at scaling up the process of making VoC.

In order to address the challenges of making structurally correct, multicellular VoC while using a simple method with the aim of producing large numbers of vessels at a time, we investigated the possible use of the viscous finger patterning (VFP) technique [20,21,22]. We took advantage of the VFP process with some significant modifications to create VoC made of one or two concentric layers of vascular cells arranged around a hollow lumen. This allowed for the formation of structurally correct capillary-like blood vessels which were constituted of either only endothelial cells or of endothelial and perivascular cells. The VoC were validated on their structural properties and functional reactivity to permeability and inflammatory challenges. These approaches allowed for testing several tens of blood vessels simultaneously in standard multiwell plate format suitable for high-throughput drug screening.

## 2. Materials and Methods

### 2.1. Microfabrication

Channels were designed in two different dimensions: h0.4 mm × w0.4 mm and h0.3 mm × w1 mm channels. Channel lengths were set at 9 mm in order to correspond to the spacing of standard 96-well SBS plates. Digital files of drawings (Autodesk Fusion 360 2020, v2.0.10440) were downloaded to a Datron NEO machining center (Datron, Sevrier, France), and patterns were machined in 10 mm-thick aluminum plates to make templates for PDMS-molding of devices. Dimensions were verified by a mechanical comparator on marble and optical imaging for height and width measurements, respectively. A PDMS mixture (Sylgard 184 Silicone Elastomer Kit, Dow chemicals, Ellsworth Adhesives France, Pontoise, France) prepared with a monomer:curing agent ratio of 10:1 was poured into the molds and baked at 90 °C for 1 h. Fluidic inlets and outlets were punched with a 1.2 mm Ø biopunch, PDMS slabs, and 115 × 75 × 0.3 mm gold seal cover glasses (Thermo Fisher Scientific, Illkirch, France) were then successively rinsed with ethanol, isopropanol, demineralized water, and carefully dried using a nitrogen spray gun. The PDMS surface was bonded to the glass by plasma surface activation using a Corona SB Plasma Treater (BlackHole Lab, Paris, France) for 5 to 10 min before contact. A last baking step at 90 °C for 3 h was performed to improve bonding between the surfaces. The chips were stored in sterile Petri dishes at room temperature until use.

### 2.2. Cell Culture

Primary human umbilical vein endothelial cells (HUVEC) and primary normal human lung fibroblasts (NHLF) were from Lonza (Verviers, Belgium) and cultured in EGM-2 and FGM-2, respectively, according to manufacturer’s instructions. THP-1 monocytes (ATCC, LGC Standards, Molsheim, France) were cultured in RPMI 1640 medium (Thermo Fisher Scientific) supplemented with 10% heat-inactivated fetal bovine serum (Hyclone, Thermo Fisher Scientific), 1 mM Na pyruvate, 50 µM β-mercapto-ethanol, 50 U/mL penicillin, 50 µg/mL streptomycin. HUVEC and NHLF were used between passages 1 and 7. All cells were cultured in humidified 95% air/5% CO_2_ incubators at 37 °C.

### 2.3. VFP Making of VoC

A neutralized collagen solution was prepared by mixing Cellmatrix Type I-A solution (Fujifilm WAKO, Sobioda, Montbonnot St Martin, France), Hank’s buffer 10× (Thermo Fisher Scientific) and collagen buffer (NaHCO_3_ 260 mM, HEPES 20 mM, 0.05 N NaOH) at a volume ratio of 8:1:1 on ice. VFP was performed by filling microchannels with 15 µL of collagen solution through one of the inlets and leaving the pipet-tips in place, then by injecting 2–3 µL of either cell-free, NHLF or HUVEC (30,000 cells/µL) cell suspensions in the opposite inlets. The devices were incubated at 37 °C in order to allow collagen gelation and cell adhesion when needed. After 15 min, 20 µL of culture medium were added to the inlet, and the pipet-tips were carefully removed in a twisting motion. The procedure was eventually performed a second time for double VFP, after removing the medium from the inlets.

### 2.4. Permeability Assays

VoC were injected with 20 µL thrombin (T4393, Sigma-Aldrich chimie, L’lsle-d’Abeau Chesnes, France) at the indicated concentrations in PBS and stimulated for 4–6 h after which 30 µL of 0.1 mg/mL Dextran 70 kDa-FITC or Texas-Red (Thermo Fisher Scientific) were injected. Diffusion was video recorded under fluorescent lighting using a Keyence BZ-X710 microscope. The diffusive permeability coefficient *Pd* was calculated by measuring the differences between the average fluorescence intensity in the gel and in vessel regions over time. In order to extract quantitative data from the video microcopy files, an Image J macro (FIJI software v1.53c [23]) was created to separate the vessel and the gel region. The diffusive permeability coefficient *P_d_* was then calculated using the following equation:

$$P_{d}=\frac{d}{4}\times \frac{1}{I_{0}}{\left(\frac{\partial I\left(t\right)}{\partial t}\right)}_{0}$$

where *d* is the vessel diameter, *I*_0_ is the average intensity in the vessel region, and *I*(*t*) is the average intensity in the gel region over time.

### 2.5. Adhesion of Immune Cells

Human THP1 monocytes (5 × 10^6^ cells) were incubated in the dark with 5 µM calcein-AM (Thermo Fisher Scientific) in RPMI culture medium for 30 min at 37 °C, rinsed twice in RPMI by centrifugation at 200× *g* for 5 min and resuspended at five 10^6^ cells/mL in RMPI. A total of one 10^5^ cell was injected in one of the microchannel inlet, and cells were allowed to adhere to the endothelial cell monolayer for 30 min at 37 °C. The cells were rinsed three times for 5 min in PBS and fixed in PBS, 4% paraformaldehyde for 15 min at room temperature. Fluorescent cells were visualized using a Keyence BZ-X710 microscope and the numbers of adherent cells were estimated by counting cells over the entire length of VoC.

### 2.6. Immunostainings and Microscopy

VoC were rinsed with PBS, fixed in PBS, 4% paraformaldehyde for 90 min at room temperature and blocked in PBS, 5% BSA (Sigma-Aldrich) for 1 h at room temperature. VoC were then incubated with the primary antibody in PBS, 0.5% BSA, 0.3% Triton-X100 (Sigma-Aldrich) for 2 h at room temperature, rinsed 3 times for 5 min with PBS and incubated for 1 h at room temperature with the secondary antibody in PBS, 0.5% BSA, 0.3% Triton-X100 and rinsed as above. VoC were post-fixed in PBS, 4% paraformaldehyde for 10 min, rinsed twice for 5 min with PBS and incubated in PBS, 1 µg/mL 4′,6-diamidino-2-phenylindole (DAPI, nuclear dye) for 10 min at room temperature, rinsed twice for 5 min with PBS and stored at 4 °C until observation. Primary antibodies used were anti-vascular endothelial (VE)-cadherin (sc-9989 Santa-Cruz Biotechnologies, Clinisciences, Nanterre, France, 1:250), anti-α-smooth muscle actin-FITC (Sigma-Aldrich F3777, 1:250), anti-zona-occludens 1 (Thermo Fisher Scientific 617300, 1:250). Actin filaments were stained with BODIPY Phalloïdin 558/568 (Thermo Fisher Scientific B-3475, 1:250). Images were taken using a confocal laser scanning microscope (Laser Scanning Microscope 880, LSM 880, Carl Zeiss, Oberkochen, Germany). Images were processed using ZEN 2 blue edition software (Carl Zeiss) and 3D reconstruction images were generated using the 3D viewer plugin from Image J software.

### 2.7. Reverse Transcription-qPCR

Individual VoC were lysed in 30 µL TRIzol (Thermo Fisher Scientific), cell extracts recovered and total RNA purified by phenol/chloroform extraction followed with precipitation. RNA were quantified on a NanodropOne (Thermo Fisher Scientific) and reverse transcription reactions carried out using High-capacity cDNA reverse transcription kit with RNase inhibitor (Applied Biosystems, Thermo Fisher Scientific). Quantitative PCR were performed in duplex PCR mixing cDNA with both the TaqMan FAM-labeled probe of the indicated tested gene (Thermo Fisher Scientific) and a β2-microglobulin-VIC-labeled probe, and processed for qPCR in a StepOne Plus machine. Alternatively, RT products (100 ng) were analyzed using Taqman Array custom cards (Thermo Fisher Scientific) and amplifications were performed in a QuantStudio 7 Flex machine. Cycle threshold (C_T_) values were calculated at the upper linear range of the logarithm^−2^ amplification curve using the StepOne v2.3 software or the Thermo Fisher Connect Relative Quantification application (Available online: https://apps.thermofisher.com/apps (accessed on 23 March 2022)). Data were expressed as 2^−ΔΔC^_T_ [24] where ΔC_T_ = C_T_ of transcript of interest—C_T_ of reference (B2M) measured in the same tube, and ΔΔC_T_ = mean ΔC_T_ experimental samples—mean ΔC_T_ control samples of the same experiment. Relative quantity (RQ) is 2^−ΔΔC^_T_ and transforms the logarithmic^−2^ data into decimal values.

### 2.8. Statistics

All experiments were performed at least twice in similar conditions; *n* indicates the numbers of biological replicates per shown experiment. Statistical analyses were performed using the Student–Newman–Keuls multiple comparisons test or unpaired t-test, Welch corrected, using the GraphPad Instat v3.10 software (Graphpad software).

## 3. Results

### 3.1. Microfabrication

**Figure 2:**
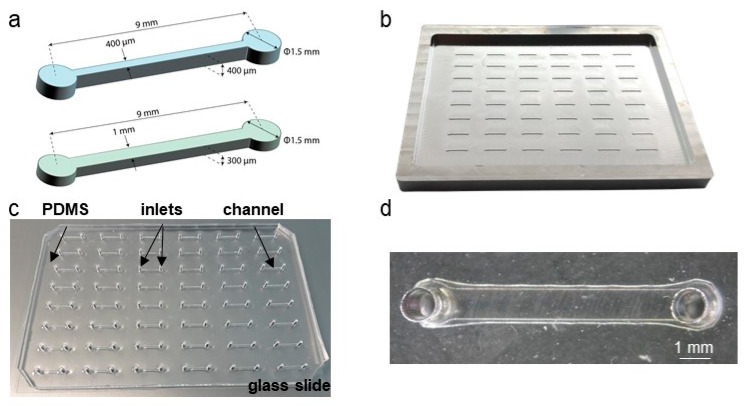
Microfluidic designs and devices. (**a**) Schematics and dimensions of the microfluidic channels. (**b**) CNC fabricated 48-channels, 96-well SBS-format aluminum mold. (**c**) 48-channels microfluidic device made of PDMS and bonded onto a 115 × 75 × 0.3 mm glass slide. (**d**) higher-power magnification of a single PDMS/glass channel.

Microchannel lengths were chosen in order for the microfluidic inlets to match the 9 mm spacing between two wells of standard 96-well SBS plates and channels produced in two dimensions: 0.4 mm (h) × 0.4 mm (w) × 9 mm (l) and 0.3 mm (h) × 1 mm (w) × 9 mm (l) (Figure 2a). Molds were carved in 1 cm-thick aluminum blocks using a computer numerical control (CNC) machine. This choice proved to be much more time- and cost-effective when compared to silicon etching or photo-lithography methods in clean-room and allowed us to create molds with excellent dimension accuracies in minutes (Figure 2b, Table 1). The aluminum plates were used as templates for PDMS-molding of devices which were then bonded to glass-plates and punched for fluidic inlets via standard methods (Figure 2c,d).

### 3.2. VFP Blood Vessel Design

**Figure 3:**
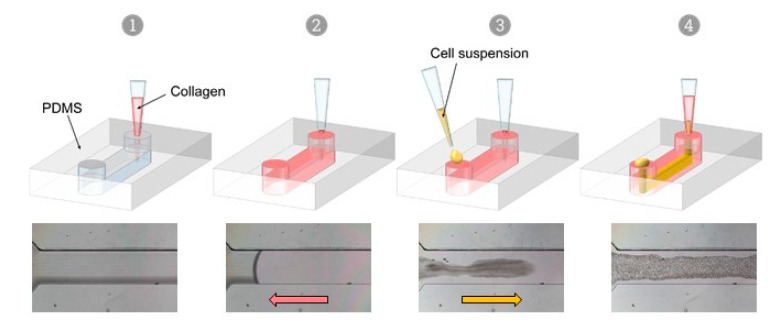
Viscous finger patterning approach. Successive steps of the viscous finger patterning technique, top; principle, bottom; phase-contrast images of VFP applied in a channel loaded with collagen (**1**, **2**), then with a cell suspension in culture medium which progresses through the collagen solution (**3**, **4**) in a finger-like shape.

VFP is a microfluidic technique that exploits the differences in viscosity between two fluids to generate a tubular scaffold. When a low viscosity fluid displaces a higher viscosity one inside a microchannel, the less viscous fluid tends to progress as a finger shape—the Saffman–Taylor finger—and to carve a distinct tubular structure through the more viscous fluid, in the middle of the microchannel [25,26]. Thus, VFP is based on passive pumping due to a pressure difference (ΔP) between the inlet and the outlet of a PDMS microchannel. The ΔP may be generated by several methods, such as applying droplets of different volumes (i.e., different internal pressures) at the inlet and at the outlet, or by creating a height difference in the liquid columns at each end of the microchannel [20]. We chose to test this technique for practical reasons because of its seeming ease of implementation. Practically, an ice-cold neutralized collagen solution is introduced using a 200 µL pipet tip into the microchannel, and the pipet tip still containing approximately 15 µL of collagen solution is left in place, lodged in the PDMS inlet so as to form a column of collagen solution above the microchannel level. Then, and instead of using a simple less viscous solution [27], a 2–3 µL droplet of high-density endothelial cell suspension in culture medium is loaded in the other inlet of the microchannel. The Saffman–Taylor finger is therefore formed by the endothelial cell suspension which progresses through the collagen-filled PDMS/glass microchannels, thus achieving the carving of the lumen and the seeding of cells in a single step. After allowing collagen reticulation and cell adhesion, non-adherent cells are rinsed from the lumens, adherent endothelial cells have lined the entire surface of the inner side of the collagen tube, and this forms a confluent monolayer after two days of culture (Figure 3, Supplementary Figure S1 and Supplementary Videos S1 and S2).

This approach allows a much a less troublesome seeding of endothelial cells than the original approach [20,22]. In particular, it does not require emptying the collagen channels prior to loading the endothelial cells, avoiding the formation of bubbles at the loading steps and also preventing the collagen gel collapse before seeding. It is also notably time saving over the initial two-steps method [20,22]. Finally, this approach does not require the use of any specific instrument besides laboratory pipettes and tips, which makes it perfectly compatible with the fabrication of several tens of vessels in a row, either manually or with pipetting robots.

In the VFP process, the diameter of the final lumen is correlated with the hydrostatic pressure applied by the less viscous solution [28]. Here, the internal lumen diameter spanned 70–80% of the dimensions of the PDMS/glass microchannels (Table 2).

**Figure 4:**
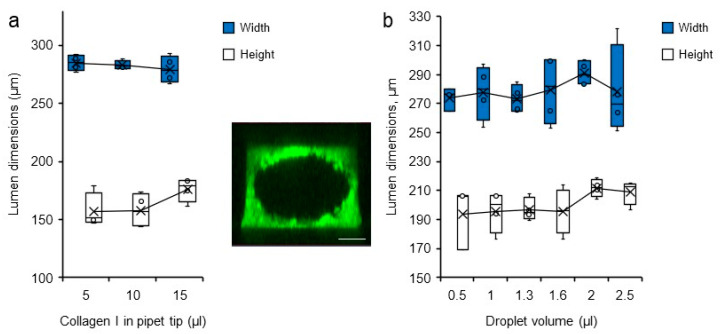
Variations in lumen dimensions. Dimensions (height and width) of the lumens measured by confocal microscopy according to (**a**) the volume of collagen I solution loaded in the pipet tip at the time of channel filling *n* = 4, and (**b**) the volume of droplets loaded into the microchannel filled with collagen solution, *n* = 4. (**insert**). Confocal microscopy maximum intensity projection 3D reconstruction of a representative collagen I/BSA-FITC channel used for measurements, bar = 200 µm.

In attempts to modify the dimension of the carved lumen, various volumes of collagen solution loaded into the microchannels and of droplets used for the VFP itself were tested. Lumen dimensions were visualized after collagen reticulation by staining the hydrogel with bovine serum albumin (BSA)-FITC which simply diffused into the gel. The channels were thereafter analysed by 3D confocal microscopy reconstruction. Changing the volume of collagen, and thus the collagen column height, did not affect the final diameter of the lumens. Similarly, changing the droplet volume did not significantly affect the dimensions of the lumen carved into the collagen solution either (Figure 4).

Therefore, the only efficient way to change the lumen diameter by applying VFP once in our conditions was to change the physical dimensions of the initial PDMS/glass microchannels, at the design level (Table 2). For practical conditions, we chose to use a volume of 15 µL of collagen solution for filling the microchannels and making the tip column, and a 2–3 µL volume of cell suspension for the VFP process itself.

### 3.3. Quality of the VoC Endothelium

**Figure 5:**
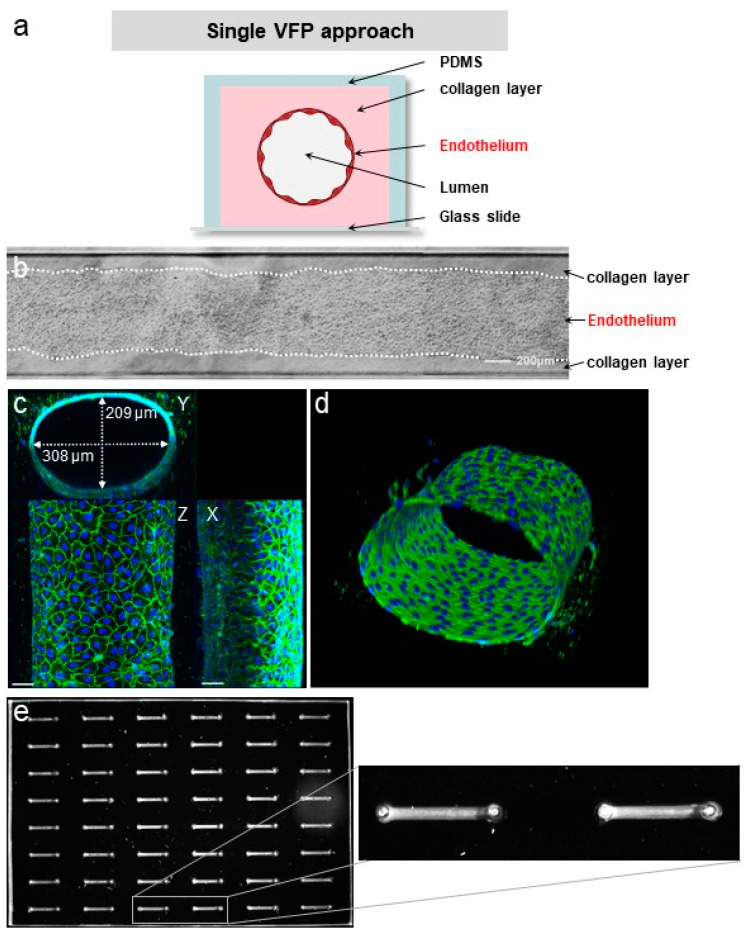
VFP making of blood vessels. (**a**) Principle of vessel construction using VFP (not to scale). (**b**) Phase contrast images of a vessel created by VFP, seeded with HUVEC, bar = 200 µm. (**c**) Confocal images of VoC after immunostaining of the nuclei (DAPI, blue) and of the adherens junction (VE-cadherin, green). Maximum intensity projections of X, Y and Z axis, bar = 50 µm. (**d**) 3D reconstruction of the VoC shown in (**c**). (**e**) left—Fluorescent imaging of a 48-channel plate where VoC were created by VFP and filled with calcein AM-labeled cells for illustration; right—image magnification of two contiguous VoC.

A structurally sound endothelium is characterized by a continuous monolayer of adjacent cells which share adherens junctions that line the cell contacts. Phase-contrast images of VoC showed a homogenous and continuous monolayer of endothelial cells (Figure 5b). In order to further assess the biological quality of the VoC, immunofluorescence staining of the endothelium-specific adherens junction molecule VE-cadherin was performed and analysed by confocal microscopy followed by 3D image reconstruction. Staining showed tubular VoC showing a typical, continuous and intercellular distribution of VE-cadherin throughout the vessel monolayer, as expected from a normal and quiescent endothelium (Figure 5c,d).

As per design, the process could be repeated on multichannel plates for the simultaneous seeding of up to 48 VoC prepared under the same conditions (Figure 5e).

### 3.4. Response to Permeability Challenge

**Figure 6:**
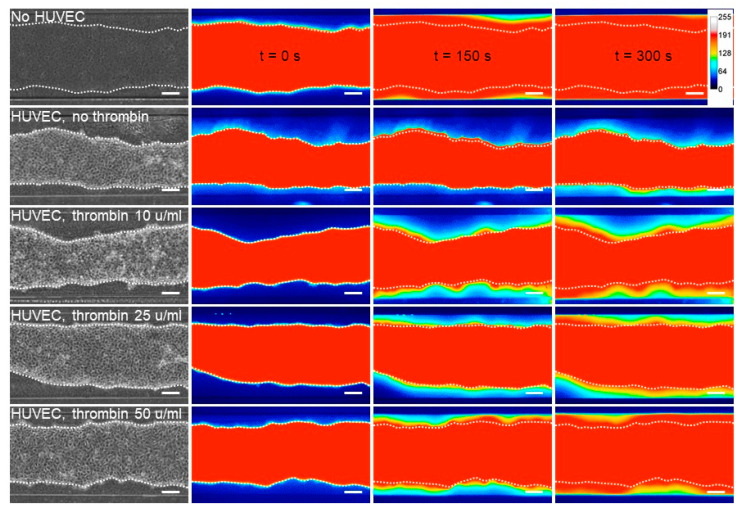
Permeability assay on VoC. Diffusion of 70 kDa dextran-FITC into the collagen layer overtime, depending on the received dose of thrombin injected. No HUVEC—collagen channel not lined with endothelial cells; HUVEC—channels lined with endothelial cells, *n* = 3 VoC per condition, bar = 200 µm. Images were extracted from video recordings at 0, 150 s or 300 s after dextran-FITC injection. Heatmap represents fluorescent intensity of 70 kDa dextran. Fifteen VoC were processed at a time in this representative experiment.

The endothelial barrier integrity is provided by the specific inter-endothelial adherens and tight junctions. The transient opening of this barrier allows the diffusion of blood borne metabolites into the surrounding tissues, a hallmark of inflammation [5]. Here, the integrity of the VoC endothelium and its responsiveness to permeability stimuli were assessed by stimulating VoC with increasing amounts of thrombin followed by dextran 70 kDa-FITC injection in the vascular lumens. Dextran diffusion through the endothelial layer and toward the collagen gel was video-recorded over 300 s. Dextran-FITC diffused at the fastest rate when the collagen lumens were not seeded with endothelial cells, whereas the presence of an endothelium markedly slowed the process (Figure 6), providing evidence that the endothelium made an efficient barrier. Furthermore, when VoC lined with endothelial cells were stimulated with increasing amounts of thrombin, ranging from 10 to 50 u/mL, there was a corresponding increase in the dextran-FITC rate of diffusion out of the lumens, showing that the endothelium was responsive to this natural vascular permeant (Figure 6).

**Figure 7:**
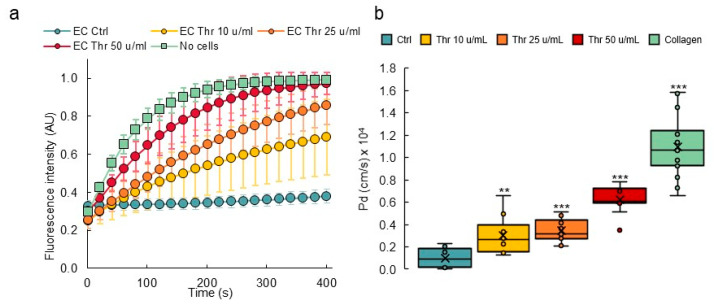
Permeability response of VoC to thrombin. (**a**) Diffusion of 70 kDa dextran-FITC into the collagen layer overtime measured from fluorescent video microscopy movies. EC—endothelial cells; Thr—thrombin. No cells: collagen channels not lined with EC. *n* = 3 VoC per conditions, 5 regions per vessel were selected to quantify the diffusion, 1 point each 10 s was extracted from the real-time 3 images/s measurements. (**b**) Calculated diffusive permeability coefficients, legend as in (**a**). *n* = 3 VoC per conditions. Five regions were selected per vessel to quantify the diffusion, ** *p* < 0.01, *** *p* < 0.001 when compared to Ctrl. Fifteen VoC were processed at a time in this representative experiment.

In order to calculate the diffusive permeability coefficients, image analyses of real-time video microscopy recordings over 5 min periods after injection of dextran-FITC were performed. For this purpose, a custom-made Image J macro was created to calculate the diffusive permeability coefficient (*P_d_*) [29] which consisted of measuring the variations of fluorescence intensities in the collagen gel areas close to the endothelial monolayer (Figure 7a, Supplementary Figure S2). The results showed that collagen lumens not lined with endothelial cells had the highest calculated *P_d_* (1.09 × 10^−4^ cm/s), whereas the presence of a confluent endothelium lowered the coefficient to 0.10 × 10^−4^ cm/s. Treating endothelial cell-lined vessels with increasing amounts of thrombin induced a proportional increase in permeability, as seen from the calculated diffusive coefficients (Figure 7b). As such, this method allowed us to monitor the permeability of dyes in real time and to precisely quantify the diffusion parameters at any given time point during the experiment.

**Figure 8:**
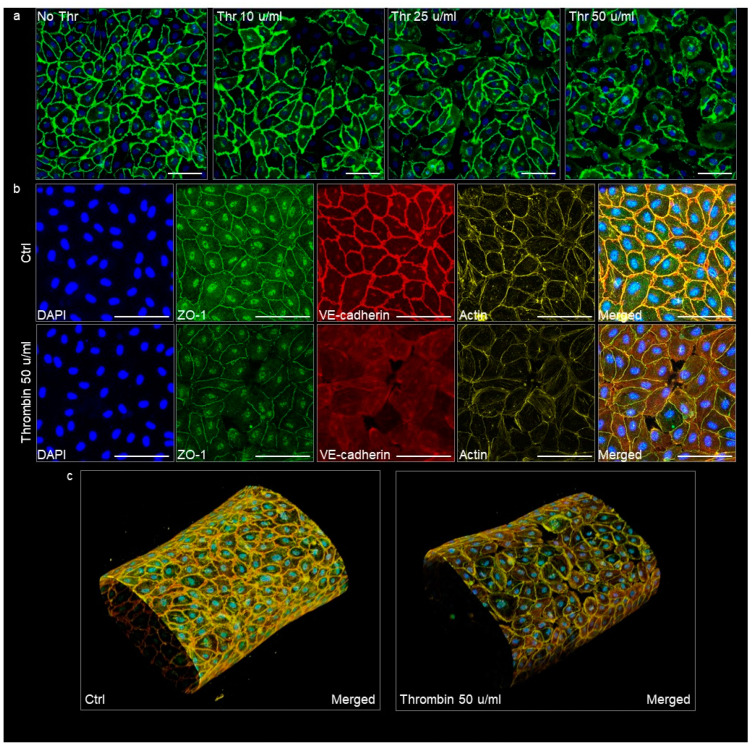
Confocal images of VoC immunostained after the permeability assays. (**a**) VoC treated with increasing amounts of thrombin were immunostained for the adherens junction molecule VE-cadherin (green) and nuclei were stained with DAPI (blue). (**b**) VoC treated or not with 50 µ/mL thrombin were stained for nuclei (DAPI), the tight-junction molecule ZO-1, the adherens junction molecule VE-cadherin, and for actin. Maximum intensity projection of the *z*-axis. Ctrl—untreated VoC; Thr—thrombin, bars = 100 µm. (**c**) 3D reconstructions of VoC, merged staining as in (**b**).

The VoC used in permeability assays were immunostained afterwards in order to visualize the endothelium adherens and tight junctions as relevant indicators of its integrity. Untreated vessels showed a homogenous and continuous lining of endothelial cells with VE-cadherin, illustrating an intact adherens junction in good correlation with the low diffusive coefficients measured in this condition. VoC which had been treated with increasing amounts of thrombin showed a corresponding increase in the disruption of adherens junctions, up to a point where open gaps were observed in the cell monolayers treated with 50 μ/mL thrombin (Figure 8a). The treatment with thrombin similarly disrupted the organization of the tight junctions, as observed after staining for zonula occludens-1 (ZO-1), and it also affected the cortical actin network organization (Figure 8b,c). These observations correlated with the extreme diffusive permeability coefficients calculated with these VoC.

### 3.5. Inflammatory Activation of Blood Vessel Endothelium

**Figure 9:**
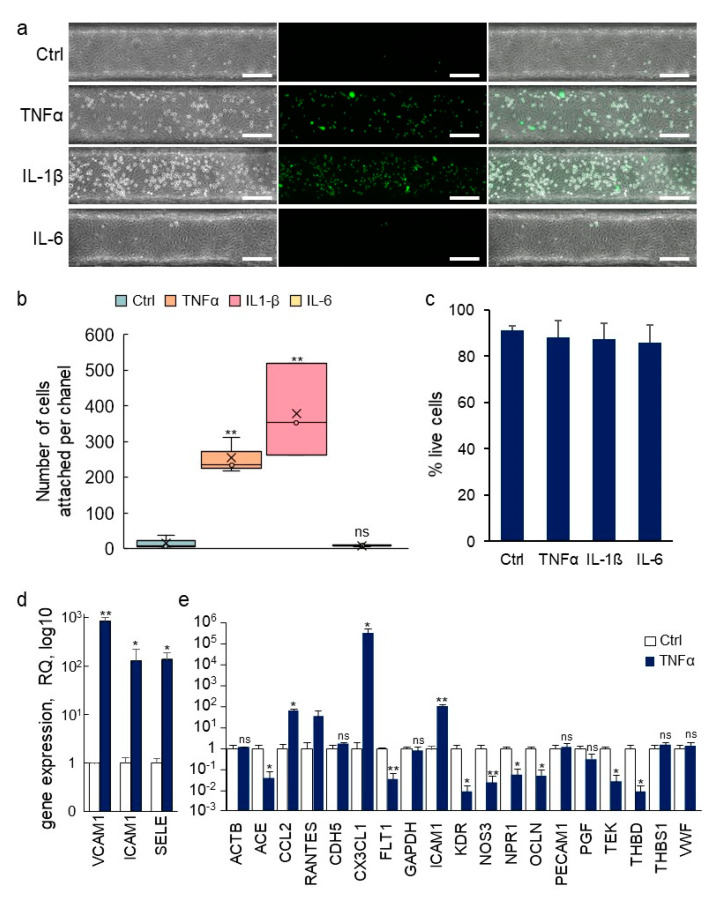
Immune activation of VoC. (**a**) VoC were stimulated or not with TNFα (0.57 nM), IL-1β (0.59 nM), or IL-6 (0.48 nM) for 4 h, then, calcein AM-labeled THP-1 monocytes (green) were injected in the lumens and allowed to adhere onto the luminal face of the endothelium. Left—phase contrast; middle—fluorescent imaging; right—merged images, bar= 200 µm. (**b**) Quantification of the numbers of immune cells attached to the vessel walls along the entire channels, *n* = 3. (**c**) Percentage of living endothelial cells after treatments with cytokines as in (**a**). (**d**) RT-qPCR array analysis of gene expression in endothelial cells recovered from VoC following stimulation or not with TNFα (0.57 nM) for 4 h. (**e**) Taqman array card analysis of expression of genes following extraction of single vessels-on-chips, a selection of genes is presented, *n* = 4, * *p* < 0.05, ** *p* < 0.01; ns—nonsignificant, compared to Ctrl.

Another fundamental role of the vascular barrier is an active involvement in the extravasation of blood-borne immune cells and their infiltration into inflamed tissues [30]. In order to assess whether the fabricated blood vessels would respond to inflammatory signals by undergoing such activation, the pro-inflammatory cytokines tumor necrosis factor-α (TNFα), interleukin (IL)-1β or IL-6 were injected into the lumens of established VoC. After this treatment, calcein AM-labeled THP-1 human monocytes were injected intraluminally to assess whether the treatment had activated the endothelium and would induce the adhesion of the immune cells to the endothelial cells. Adhesion of THP-1 monocytes to nonstimulated VoC was barely detectable (Ctrl 16 ± 18 cells/vessel, Figure 9a,b). On the other hand, stimulation of the VoC with TNFα or IL-1β resulted in a large increase in the numbers of immune cells adhering to the endothelial surface, reaching 255 ± 50 and 378 ± 130 cells/vessels, respectively. Minimal adhesion of immune cells was observed when blood vessels were stimulated with IL-6 (8 ± 3 cells/vessel), which was used as an inactive interleukin control because HUVEC do not express the IL-6 receptor gp80 [31]. Of note, treatments with cytokines in these conditions did not yield any significant differences in endothelial cell death as cell viability remained very high, above 85% in all VoC (Figure 9c).

Overall, the process of establishing VoC constituted solely of endothelial cells allowed for the formation of a normal and quiescent endothelium, such as those found in non-inflamed tissues. These VoC were yet well responsive to natural pro-inflammatory cytokines, allowing them to mimic an inflamed vascular situation when needed.

### 3.6. Transcriptomic Analyses of Single Vessels

The prospect of making molecular analyses of biological processes which occur in organ-on-chip devices is interesting as it provides additional information on the molecular pathways involved. However, this approach is generally limited by the small number of cells available in each device and by the limited number of devices used at one time. Here, the dimensions of the VoC allowed it to recover enough cell lysate from each single VoC for gene expression analyses. In addition, because several vessels could be treated in similar conditions at the same time, the analysis of multiple biological replicates from the same experiments was possible. Endothelial cell extracts were prepared from one vessel at a time, total RNA purified and gene expression was assessed using Taqman RT-qPCR. As an example of this process, expression of intercellular adhesion molecule-1 (ICAM-1), vascular cell adhesion molecule-1 (VCAM-1) and of E-selectin (SELE) was assessed in endothelial cells recovered from single VoC which had been stimulated or not with TNFα. These genes are known to be significantly upregulated in endothelial cells following stimulation with pro-inflammatory cytokines [2]. Here, the expression levels of SELE, ICAM-1, and VCAM-1 showed a 138 ± 51-, 130 ± 94- and 850 ± 171-fold increase following TNFα stimulation, respectively, when compared to nonstimulated vessels (Figure 9d). Furthermore, RNA extracts from single VoC could also be used to analyze the expression levels of several tens of endothelial genes using Taqman Array cards (Figure 9e). Among the genes typically up or downregulated by TNFα stimulation of HUVEC in addition to those analyzed above, the VoC showed, on the one hand, a significant increase in expression of CCL2, CX3CL1, ICAM-1 and, on the other hand, a down-regulation of ACE, FLT1, KDR, NOS3, NRP1, OCLN, TEK, and THBD, in good correlation with previous reports [32,33,34,35,36,37].

### 3.7. Establishing Multilayered Vessels by the Use of Double VFP

VoC made of solely endothelial cells are relevant to mimic newly formed capillaries or unstable blood vessels, such as those found in tumors [38]. However, in most organs, perivascular cells, such as pericytes in capillaries and smooth muscle cells in larger vessels, are present at the abluminal side of the endothelia where they participate in the stability and in the maintenance of the blood vessel barrier integrity, among other functions [39]. In order to mimic this situation, we aimed at seeding perivascular cells around the endothelial cells in the VoC. As a proof of feasibility, we firstly designed VoC using a double-VFP approach, which consisted of carving a channel in a collagen solution by VFP with culture medium and letting the collagen harden. Then, a second collagen solution was injected into the channel, also via VFP, and the lumen was then carved using an endothelial cell suspension. This resulted in the formation of two concentric layers of collagen, the second one of which was lined with endothelial cells (Supplementary Figure S3). As expected, narrower vascular channels than those made with single VFP were formed and, combining the use of 0.4 mm- or 1 mm-wide channels with this double VFP approach, allowed us to create vessels of diameters ranging from approximately 270 µm to 500 µm (Supplementary Table S1).

**Figure 10:**
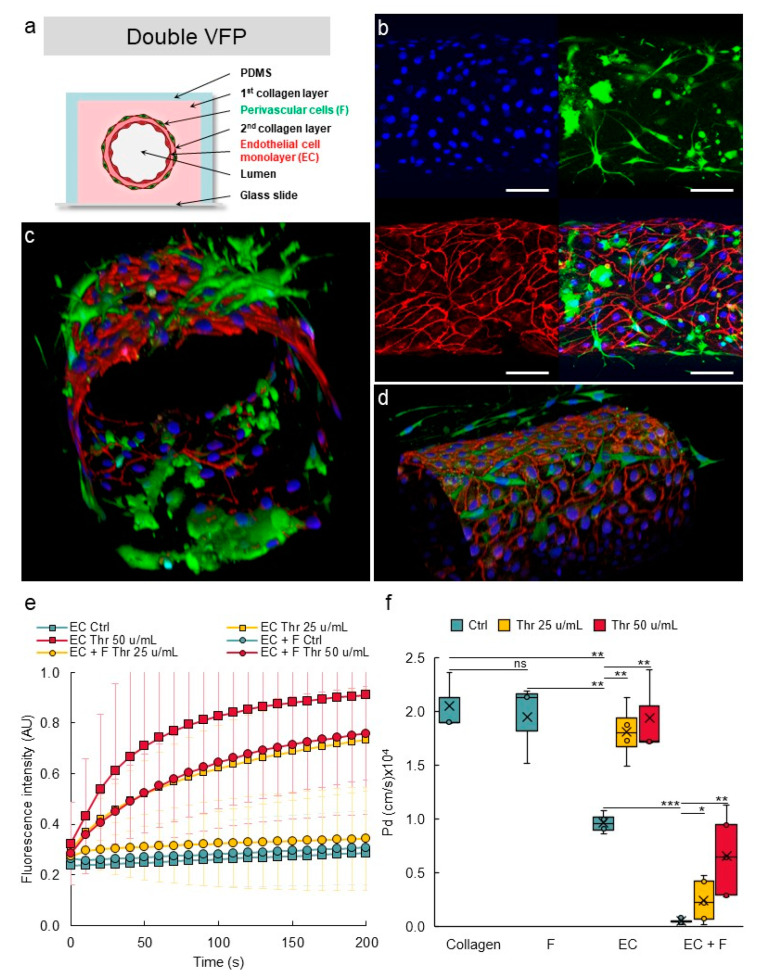
Design of multilayered VoC. (**a**) Principle of multilayer VoC construction using double VFP (not to scale). (**b**) Confocal images of VoC immunostained for endothelial cells (VE-cadherin, red), fibroblasts (green), nuclei (DAPI, blue), maximum intensity projections of the Z-axis of the separate fluorescence channels and merge, bar = 100 µm. (**c**,**d**) 3D reconstructions of multilayered VoC. (**e**) Diffusion of 70 kDa dextran-FITC into the collagen layer overtime; data are extracted from video recordings; EC—endothelial cells; F—fibroblasts; Thr—thrombin. No cell: collagen channels not lined with EC. *n* = 3 VoC per condition; diffusion was quantified over the total length of the vessel; 1 point each 10 s was extracted from the real-time 3 images/s measurements. (**f**) Calculated diffusive permeability coefficients depending on the conditions. Collagen: channel not lined with EC. *n* = 3 VoC per condition, five regions were selected per vessel to quantify the diffusion; VoC were established five days before performing the permeability assay, * *p* < 0.05, ** *p* < 0.01, *** *p* < 0.001; ns—nonsignificant when compared to Ctrl. Twenty-four VoC were processed at a time in this representative experiment.

After setting up the double VFP conditions, this approach was performed using the two types of vascular cells. Normal human lung fibroblasts (NHLF) were used as perivascular cells, as these cells were previously shown to behave as perivascular cells in vitro [40,41]. A first VFP was performed using a solution of collagen poured into the PDMS/glass channels and a suspension of NHLF in culture medium as the less viscous solution. After gelling of this first collagen layer which was therefore seeded with NHLF, a second layer was created by injecting again a collagen solution, immediately followed by VFP using an endothelial cell suspension (Figure 10a). This resulted in the deposition of a thin collagen layer on top of the NHLF layer and the seeding of endothelial cells at the same time. Using this original approach, the two principal layers of cells which make blood vessels were established in their correct and respective setting, as in natural vessels (Figure 10a). The endothelial cells formed a homogenous monolayer lining the lumen and immunostaining for VE-cadherin showed the expected typical pattern of continuous adherens junctions between cells. Importantly, the NHLF were established around the endothelium tube with no mixing with the endothelial layer. Furthermore, NHLF adopted a stellate shape and projected multiple finger-like filipodia extending through the thin collagen layer to make direct contacts with endothelial cells. This typical morphology perfectly matched that observed for pericytes in natural blood vessels (Figure 10b–d).

In order to assess whether the presence of perivascular cells improved the vascular barrier properties, VoC composed of either or of both HUVEC and NHLF were tested in permeability assays. A maximal diffusive permeability coefficient was measured when no cells were added to the collagen lumen, as above. Adding only NHLF did not improve the barrier properties of the “vessel” which remained leaky because lumens were constituted of solely collagen (Figure 10e,f), suggesting that NHLF did not provide per themselves a physical barrier to the diffusion of dextran-FITC outside of the vessel lumen. VoC made of only endothelial cells established as a single monolayer created a barrier which allowed a slow but measurable spontaneous diffusion of dextran-FITC into the collagen gel overtime. Addition of thrombin disrupted this barrier and allowed the dextran to diffuse at significantly much higher rates (Figure 10e,f). On the other hand, addition of NHLF (1%) around the endothelial monolayer resulted in a marked tightening of the barrier properties, as the spontaneous diffusion of dextran-FITC could not be detected anymore over the time-course of the experiments. These multilayered VoC were also much less responsive to thrombin. A treatment with thrombin at 25 u/mL or at 50 u/mL resulted in a significantly slower diffusion of dextran-FITC compared to the effects observed using similar doses in the blood vessels solely composed of endothelial cells (Figure 10e,f).

### 3.8. Perivascular Cells Participate in the Tightness of the Vascular Barrier

**Figure 11:**
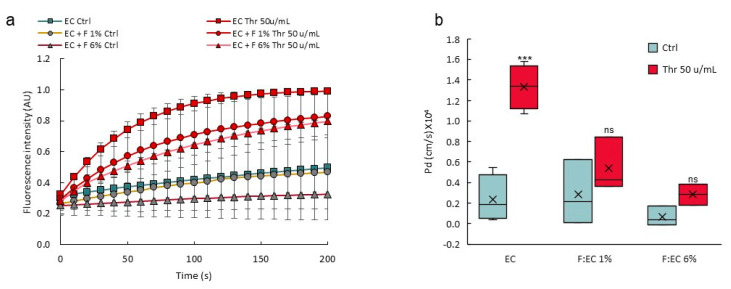
A higher perivascular:endothelial cell ratio tightens the vascular barrier. (**a**) Diffusion of 70 kDa dextran-FITC into the collagen layer overtime; data are extracted from video recordings; *n* = 3 VoC per condition; 1 point each 10 s was extracted from the real-time measurements (3 points/s). (**b**) Calculated diffusive permeability coefficients, *n* = 3 VoC per condition. EC—endothelial cells; F—fibroblasts; Thr—thrombin, percentages refer to the F:EC ratio at seeding time, vessels were established eight days before performing the permeability assay *** *p* < 0.001; ns—nonsignificant compared to Ctrl. Eighteen VoC were processed at a time in this representative experiment.

VoC were made using different NHLF:HUVEC (F:EC) ratio at seeding time and challenged for permeability response after stimulation with thrombin. There was a significant and dose-dependent decrease in the response of the vascular barrier to thrombin when using a 1% or a 6% F:EC ratio, compared to the control vessels without fibroblasts (Figure 11), showing that a higher F:EC ratio had a positive effect on the VoC barrier tightness.

## 4. Discussion

The present work was based on the design of blood vessels-on-chip using an optimized VFP approach. This allowed to create initial vessels composed of endothelial cells which showed good functionalities in terms of permeability and inflammatory activation. A double VFP approach was used to create more complex and structurally correct vessels by consecutively seeding perivascular and then endothelial cells in concentric and distinct layers. The engineered VoC showed well-structured endothelial cell adherens and tight junctions and were reactive to immune activation and to permeability agonists. The addition of perivascular cells greatly improved the VoC barrier functions. In addition, a permeability assay was designed, and a method was set up to calculate diffusive permeability coefficients from video recordings of the diffusion of fluorescent dextran. The devices were shown to be compatible with the recovery and isolation of nucleic acids from each single vessel for transcriptomic analyses. Furthermore, the design allowed the making and use of tens of vessels in parallel, and the vessels could be challenged with biological replicates of different conditions, including positive and negative controls at the same time, making the process compatible with medium- to high-throughput screening.

The most significant improvement over existing methods for making blood vessels-on-chip is that our approach allows for the proper establishment of the two main vascular cell types in circular layers, like in natural blood vessels. Bringing perivascular cells in close vicinity of endothelial cells but not mixed with them is an important issue when making VoC. Perivascular cells are located on the abluminal side of endothelial cells in natural blood vessels. They produce survival, differentiation factors, a favorable matrix environment to endothelial cells, and they play a crucial role in the maintenance of quiescent and stable blood vessels in vivo [42]. Earlier VoC designs using perivascular components had either randomly seeded perivascular cells into the whole volume of the hydrogel surrounding the endothelial layers [16,17] or had created mixed layers of endothelial and perivascular cells [18]. In the first case, the models did not reproduce the close apposition of the two cell types. In the second approach, the mixed endothelial/perivascular cell layer compromised the required continuity and integrity of the endothelial monolayer. Our approach brings both cell layers in close proximity but as distinct sheets, keeping them separated by a thin layer of collagen to better mimic the structuration of natural blood vessels.

In blood vessels, the presence of supporting pericytes prevents leakage through the vessel wall and reduces the permeability of the endothelium, whereas defective pericyte coverage induces a poorly organized and leaky vasculature [43]. In the present VoC, lung fibroblasts appeared to functionally replace pericytes as they markedly increased the tightness of the vessel barrier and lowered the response to thrombin. Choosing NHLF as a source of perivascular cells in making VoC appeared pertinent from a biological point of view, firstly because pericytes mostly derive from the mesothelium during embryonic development in vivo [42]. Second, fibroblasts cells were shown to be essential to blood vessel lumen formation in vitro, due to the production of matrix proteins [44]. In addition, lung fibroblasts have historically demonstrated their pertinence in VoC designs for their capacity to support tube-like self-assembly of endothelial cells and to increase vessel barrier functions [40,41]. Finally, a recent study using fibroblasts from various origins showed that fibroblasts from lung tissue, such as those used here, performed best as supporting cells in 3D vascular models [45]. In our conditions, the NHLF adopted a typical stellate-shape pericyte morphology, extending fiber-like protrusions and making contact with endothelial cells, as in natural capillary vessels [43]. Whether the NHLF used here produced pericyte factors and matrix molecules that stabilized the endothelial junctions and whether the presence of endothelial cells engaged the NHLF in a pericyte-like phenotype is not known at this time but would certainly be worth investigating. The perivascular:endothelial cells ratio varies in vascular beds, from 1:1 in the retina to 1:100 in skeletal muscles [43], although these exact numbers remain controversial [42]. There was definitely a marked increase in the integrity of the vascular barrier in the VoC when the NHLF:HUVEC ratio was increased, and this is in good accordance with fact that pericyte deficiency caused increased brain vessel permeability [46,47] and a poorly organized and leaky vasculature in tumors [48].

VoC models need to be functionally validated by showing proper biological responses during typical vascular processes. With regards to the natural endothelium, the monolayer integrity is mainly due, first, to the proper adhesion of endothelial cells to the basal lamina and, second, to the formation of adherens and tight junctions which form a continuous barrier between endothelial cells [3]. Adherens junctions are critical to the maintenance of the vascular barrier and any disruption of these junctions leads to vessel permeability via the opening of intercellular spaces and leakage of large molecular weight plasma components into the adjacent tissues [30]. In blood vessels, VE-cadherin is the main structural component involved in the formation and maintenance of the adherens junctions. Interfering with the homophilic molecular interactions between VE-cadherin molecules on adjacent endothelial cells leads to vascular permeability [49]. It is therefore expected that VoC display proper adherens junctions and be responsive to natural permeability agonists such as thrombin. In the present VoC, endothelial cells lining the inner side of the lumens showed a typical continuous distribution of VE-cadherin at cell–cell junctions, indicating a properly established confluent monolayer such as found in vivo in quiescent blood vessels [3]. The stimulation of endothelial cells with thrombin is known to alter the localization of VE-cadherin, induce its dissociation from β-catenin, disrupt the adherens junctions and open gaps between endothelial cells [50]. In the present study, the VoC were fully responsive to thrombin which induced a dose-dependent loosening of the inter-endothelial adherens junctions and the redistribution of VE-cadherin in similar ways. Furthermore, the tight junctions were also affected by the thrombin treatment, indicating that the vessels-on-chip were functional with regards to permeability. The tight-junction adaptor protein ZO-1 is important for the establishment of the vascular barrier. ZO-1 is necessary to the correct development of blood vessels during embryogenesis [51] and its defects disrupt the endothelial tight junction. More importantly, ZO-1 also regulates the integrity of the adherens junction via the recruitment of mechanoadapters to the VE-cadherin complex [52].

Another essential biological validation of functionality is the activation of the VoC following stimulation with pro-inflammatory cytokines. This is a classical, essentially endothelial-mediated response which was, again, well reproduced in the present settings. At the functional level, a marked increase in the number of leukocytes adhering onto the luminal side of the VoC endothelium was observed following stimulation with TNFα or IL-1β. At the gene expression level, this corresponded to a significant increase in the expression levels of three of the main leukocyte adhesion receptors expressed by endothelial cells under such conditions. The variations in expression levels corresponded to those classically observed in HUVEC, ranging from ten- to thousand-fold increases following TNFα stimulation [53]. This analysis was made possible by the fact that nucleic acids could be easily recovered from each individual vessel and purified separately. Every vessel could be analyzed individually for assessing the expression levels of hundreds of genes with great accuracy using Taqman qPCR technology. This was a particular improvement over more classical settings which required the physical destruction of the devices with high risks of material degradation and which generally failed to provide enough material from single chips.

One of the main advantages of our manufacturing technique is that it allows for the simultaneous design of 48 VoC on the same device, a scale which is almost impossible to reach with other on-chip technologies using sacrificial templates, bioprinting or laser degradation [54]. On the other hand, the process of formation of the vessels via VFP did not allow for a very tight control of their geometry. The vessels show some irregularities in diameter along their length and are not perfectly cylindrical, such as those made via the use of templates [16,55,56]. While this is not an issue when studying vascular barrier functions, it may lead to inconsistencies when studying flow-related aspects that depend on homogenous and well-standardized vessel dimensions. Indeed, making circular vessels in 3D settings is important for flow conditions because round channels allow for better distribution of shear-stress forces than rectangular channels [57]. Therefore, 3D circular vessels reproduce better the natural situation with regards to fluid mechanics and tissue exposure to physical forces under perfusion. There is still a need for improving the consistency and reliability of the process of making the vessels before considering such studies. Because our devices are designed in the multiwell plate SBS format, we are currently developing an automated approach for the fabrication of VoC using a customized open-source laboratory robot. Automation would actually further increase the reproducibility of the process compared to manual pipetting, save time and probably improve the levels of control of the experiment because all the parameters will be more tightly regulated (time of delivery, volume, and speed of injection).

In conclusion, the VFP approach refined here allows us to meet the initial challenges of making structurally correct, mono- and multicellular, biologically valid VoC using a simple method, and permits the production of large numbers of vessels at a time for medium- to high-throughput analyses.

## Figures and Tables

**Table 1:** Dimensions of the designs and measures of the machined molds.

Microchannel Design	Measured Height	Measured Width
0.4 mm (h) × 0.4 mm (w)	395.8 ± 2.3 µm (*n* = 30)	398.0 ± 4.4 µm (*n* = 30)
0.3 mm (h) × 1 mm (w)	292.0 ± 2.6 µm (*n* = 30)	993.5 ± 7.4 µm (*n* = 30)

**Table 2:** Dimensions of the VoC lumens using VFP, depending on the channel dimensions.

Microchannel	Lumen Diameter
0.4 mm (h) × 0.4 mm (w)	351 ± 31 µm (*n* = 20)
0.3 mm (h) × 1 mm (w)	700 ± 54 µm (*n* = 35)

## Data Availability

The data presented in this study are available on request from the corresponding author.

## Supplementary Materials

The following are available online at https://www.mdpi.com/article/10.3390/biomedicines10040797/s1, Figure S1: Making of VoC—details, Figure S2: Image analysis procedure, Figure S3: Double VFP making of blood vessels, Table S1: Dimensions of the VoC lumens using double VFP, Video S1: Endothelial cell suspension carving through the collagen solution by VFP, Video S2: Detailed VFP procedure.
