# Supplementary material for: Multi-Layered Human Blood Vessels-on-Chip Design Using Double Viscous Finger Patterning
Source: Biomedicines. 2022 Mar 29;10(4):797. doi: 10.3390/biomedicines10040797 (PMC9027030; doi:10.3390/biomedicines10040797)
Supplement: Supplementary file 1 [file biomedicines-10-00797-s001.zip › Suppl Figure S1.pdf]

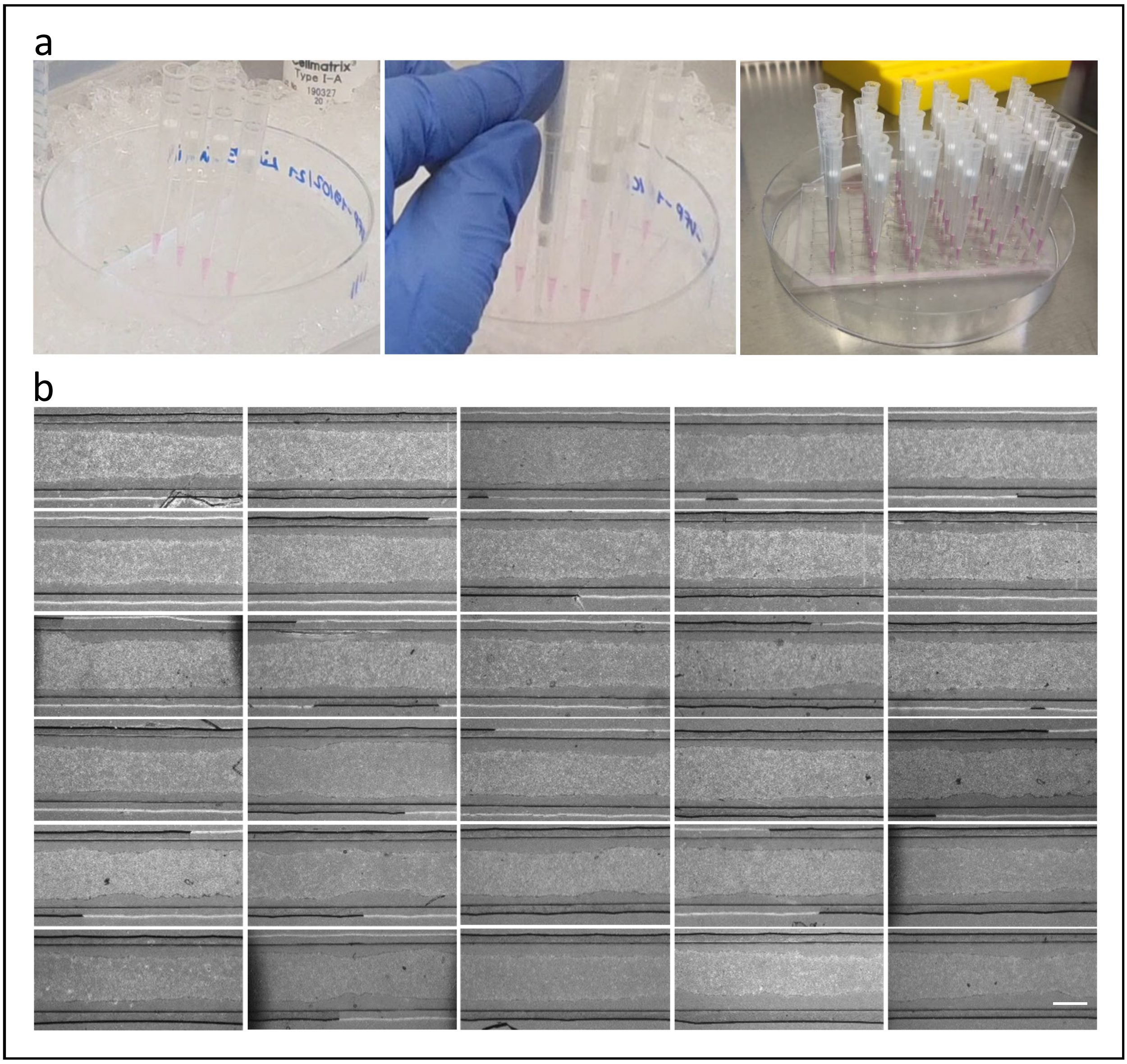

Supplementary Figure S1. Making of VoC - details. (**a**) left: the collagen solution is poured into the microchannels and tips are left in place to form a column, middle: droplets of cell suspension are loaded in the opposite inlet while the devices are still on ice to prevent collagen reticulation, right: the devices are ready for incubation at 37° C for collagen reticulation. (**b**) 30 individual vessels were created at the same time in one representative experiment and visualized by phase contrast microscopy, bar = 500 µm.
