# Supplementary material for: Multi-Layered Human Blood Vessels-on-Chip Design Using Double Viscous Finger Patterning
Source: Biomedicines. 2022 Mar 29;10(4):797. doi: 10.3390/biomedicines10040797 (PMC9027030; doi:10.3390/biomedicines10040797)
Supplement: Supplementary file 1 [file biomedicines-10-00797-s001.zip › Suppl Figure S3.pdf]

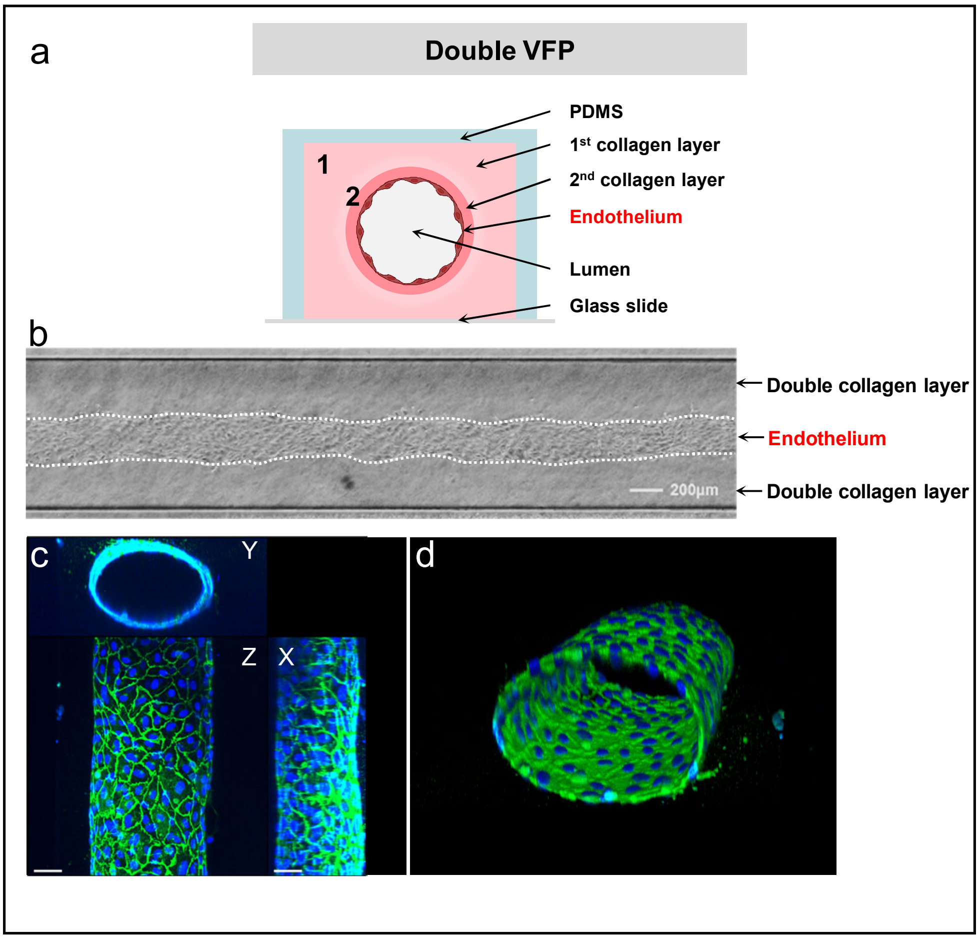

Supplementary Figure S3. Double VFP making of blood vessels. (**a**) Principle of vessel construction using double VFP (not to scale). (**b**) Phase contrast images of vessels created by double VFP, seeded with HUVEC, bar = 200 µm. (**c**) Confocal images of engineered vessel inside a 400 µm-wide microchannel using double VFP (bottom) after immunostaining of the nuclei (DAPI, blue) and of the adherens-junction (VE-cadherin, green). Maximum intensity projections of X, Y and Z axis, bar = 50 µm. (**d**) 3D reconstruction of the vessels shown in (**c**).
