# Supplementary material for: Multi-Layered Human Blood Vessels-on-Chip Design Using Double Viscous Finger Patterning
Source: Biomedicines. 2022 Mar 29;10(4):797. doi: 10.3390/biomedicines10040797 (PMC9027030; doi:10.3390/biomedicines10040797)
Supplement: Supplementary file 1 [file biomedicines-10-00797-s001.zip › Suppl Table S1.pdf]

Microchannel	Lumen diameter
0.4 mm (h) x 0.4 mm (w)	266 ± 54 μm (n = 17)
0.3 mm (h) x 1 mm (w)	505 ± 84 μm (n = 28)

Supplementary Table S1. Dimensions of the VoC lumens using double VFP, depending on the dimensions of the channel used.
