# Supplementary figures and images for: Multi-Layered Human Blood Vessels-on-Chip Design Using Double Viscous Finger Patterning
Source: Biomedicines. 2022 Mar 29;10(4):797. doi: 10.3390/biomedicines10040797 (PMC9027030; doi:10.3390/biomedicines10040797)

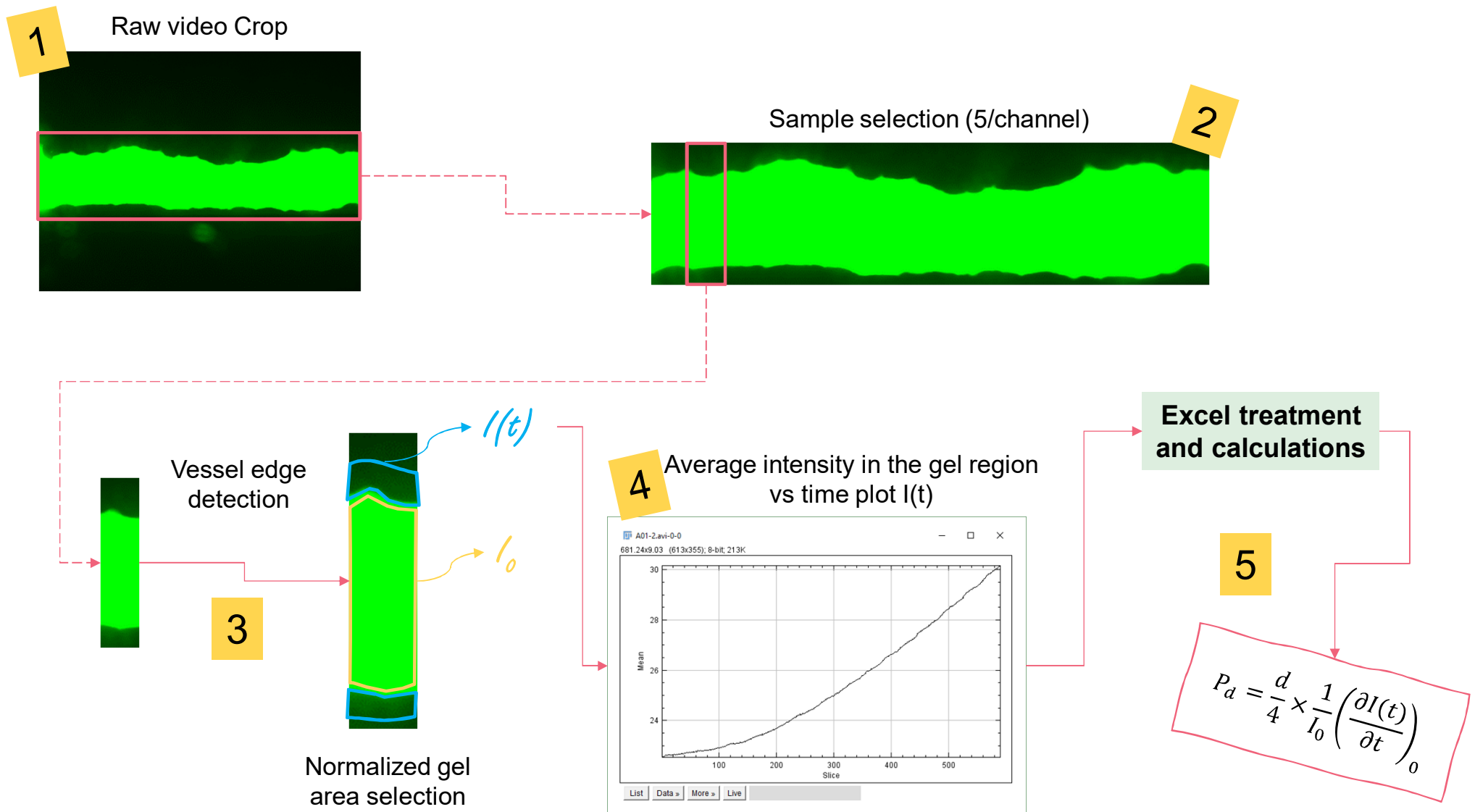

Supplementary Figure S2. Image analysis procedure.

Supplement: Supplementary file 1 [file biomedicines-10-00797-s001.zip › Suppl Figure S2.pdf]
